# Global distribution of epidemic-related Shiga toxin 2 encoding phages among enteroaggregative *Escherichia coli*

**DOI:** 10.1038/s41598-020-68462-9

**Published:** 2020-07-16

**Authors:** Keiko Kimata, Kenichi Lee, Masanori Watahiki, Junko Isobe, Makoto Ohnishi, Sunao Iyoda

**Affiliations:** 1 Department of Bacteriology, Toyama Institute of Health, Toyama, 9390363 Japan; 2 Department of Bacteriology I, National Institute of Infectious Diseases, Tokyo, 1628640 Japan

**Keywords:** Bacterial evolution, Bacterial genomics, Microbiology, Policy and public health in microbiology, Bacterial infection

## Abstract

Since the Shiga toxin-producing enteroaggregative *Escherichia coli* (Stx-EAEC) O104:H4 strain caused a massive outbreak across Europe in 2011, the importance of Stx-EAEC has attracted attention from a public health perspective. Two Stx-EAEC O86 isolates were obtained from patients with severe symptoms in Japan in 1999 and 2015. To characterize the phylogeny and pathogenic potential of these Stx-EAEC O86 isolates, whole-genome sequence analyses were performed by short-and long-read sequencing. Among genetically diverse *E. coli* O86, the Stx-EAEC O86 isolates were clustered with the EAEC O86:H27 ST3570 subgroup. Strikingly, there were only two loci with single nucleotide polymorphisms (SNPs) between the Stx2a phage of a Japanese O86:H27 isolate and that of the European epidemic-related Stx-EAEC O104:H4 isolate. These results provide evidence of global distribution of epidemic-related Stx2a phages among various lineages of *E. coli* with few mutations.

## Introduction

Enteroaggregative *Escherichia coli* (EAEC) is a subgroup of diarrhoeagenic *E. coli* that shows an aggregative adherence pattern to epithelial cells^1^. EAEC is a common cause of acute and persistent diarrhoea in both children and adults worldwide. EAEC infections are most frequently reported as self-limiting and result in mild symptons^2^; however, Shiga toxin (Stx)-producing EAEC O104:H4 caused a massive outbreak among European countries in 2011. A total of 3,842 confirmed cases, including 845 haemolytic-uremic syndrome (HUS) cases and 54 deaths, were reported^3^. The epidemic showed that EAEC has the potential to become highly virulent through the acquisition of a Stx2 phage. Besides the epidemic in Europe, several cases of Stx-EAEC from HUS patients have been reported, including strains of the following serotypes: O111:H2^4^, O86:HNM^5^, O111:H21^6^, and O59:HNM^7^.

Stxs are a group of AB5 protein toxins that inhibit protein synthesis in eukaryotic cells^8^. Shiga toxins from *E. coli* are classified into two major types: Stx1 and Stx2. These Stxs can be further subdivided into several subtypes^9^. Among the subtypes, Stx2a and Stx2d showed higher potency in cultured cells and a mouse model compared to Stx1^10^. Additionally, epidemiological studies have shown that *E. coli* carrying the *stx2a* operon are more likely to be involved in severe disease^11^. The *stx* genes are encoded in Lambda-like lysogenic phages. Therefore, Stx-encoding phages can be transferred horizontally, and phages have been found in various lineages of *E. coli* though rarely in other Enterobacteriaceae^12,13^.

A subset of *E. coli* producing Stx (or carrying the *stx* gene) is called Shiga toxin-producing *E. coli* (STEC). STEC are important foodborne pathogens that cause enteritis, bloody diarrhoea, and often fatal HUS worldwide^14^. As more than 3,000 cases of infection have been reported annually in Japan^15^, national surveillance has been performed by molecular typing methods, including multilocus variable-number tandem repeat analysis, pulsed field gel electrophoresis, and whole-genome sequencing (WGS), since 1996^16–19^. From this surveillance, two isolates of Stx-EAEC serogroup O86 from bloody diarrhoea or HUS, and two STEC O86 isolates from asymptomatic carriers were reported by 2017; however, there is no known epidemiological link or genetic information shared between the isolates. In this study, to elucidate the genomic characteristics and phylogenetic lineage of STEC O86 isolated in Japan in the last 20 years, including two Stx-EAEC, the draft genomes were analysed and compared with published WGS data of various *E. coli* strains.

To obtain the information of the most important virulence determinants, Stx2a phages and virulence plasmids, the complete genomes of Stx-EAEC O86 isolates were determined by long read sequencing. Comparative genome analyses revealed that the Stx2a phage of one Stx-EAEC O86 isolate was nearly identical to that of the European Stx-EAEC O104:H4 strain, suggesting the global distribution of the epidemic-related Stx2a phage.

## Results

### Characterization of phylogeny and pathogenic potential of Japanese STEC O86 isolates by WGS analyses using draft genome sequences

From 1999 to 2017, four STEC O86 isolates were detected by national surveillance (Table 1). To infer the phylogenetic relationships and characterize the virulence profiles of the isolates, WGS analyses of these isolates was performed and data from public databases were analysed. WGS data from a public database (EnteroBase, https://enterobase.warwick.ac.uk/species/index/ecoli) were collected from international isolates of *E. coli* O86, Stx-EAEC, and other pathogenic or commensal *E. coli* strains derived from humans and animals, as shown in Supplementary Table S1. At the time of writing, three Stx-EAEC isolates, other than O86 and O104:H4, had been reported to cause HUS as follows: O111:H2^4^, O111:H21^6^, and O59:HNM^7^. Among them, a draft genome of an O111:H21 isolate from the UK was available. This isolate was used for phylogenetic analysis and virulence factor comparison. For O111:H2 isolates from France, only the Stx2 phage sequence was available, and this was used for phage comparison as described below.

**Table 1:** Strains used in this study.

ID	BioSample	Serotype^a^	Year	Symptom	Pathotype	Phylogenetic group	*stx*
JE86-ST02	SAMD00137267	O86:H27	1999	HUS	Stx-EAEC	B1	2a
JE86-ST03	SAMD00137268	O86:H32	2007	Asymptomatic	STEC	A	2e
JE86-ST04	SAMD00137269	O86:H51	2011	Asymptomatic	STEC	B1	1a
JE86-ST05	SAMD00137270	O86:H27	2014	Bloody diarrhea	Stx-EAEC	B1	2a

^a^Serotype predicted by the draft genomes. All the isolate showed non-motile phenotype.

**Figure 1:**
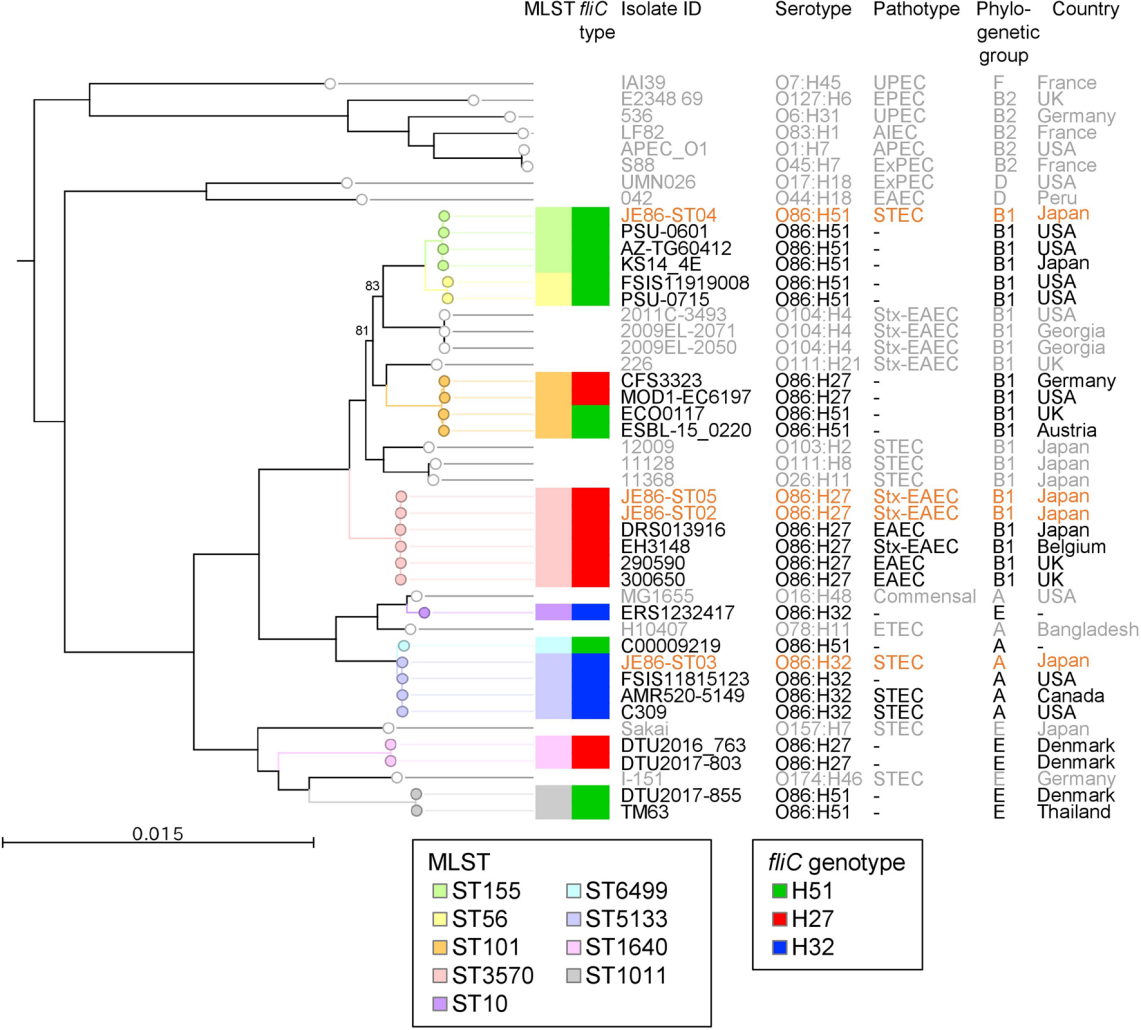
Core genome SNP-based maximum likelihood tree of *Escherichia coli* O86 and other *E. coli* serogroups. The colour of branches and nodes (open circles) represents the sequence type (ST) by multilocus sequence typing (MLST). The colour of boxes on the right represents the ST and the *fliC* genotype (in silico H type). Isolate names sequenced in this study are shown in red. Information of the non-O86 isolates was shaded. The tree was rooted by *E. albertii* 2012EL-1823B. Bootstrap values below 95 are shown at the branch. Abbreviations of the pathotypes were as follows: UPEC, uropathogenic *E. coli*; EPEC, enteropathogenic *E. coli*; AIEC, adherent invasive *E. coli*; APEC, avian pathogenic *E. coli*; ExPEC, extraintestinal pathogenic *E. coli*; EAEC, enteroaggregative *E. coli*; STEC, Shiga toxin-producing *E. coli*; Stx-EAEC, Shiga toxin-producing enteroaggregative *E. coli*; ETEC, enterotoxigenic *E. coli*; -, not available. The phylogenetic group was determined by in silico PCR.

Although all the STEC O86 in Japan was non-motile, sequencing of the flagellar gene, *fliC*, identified three H-types (H27, H32, and H51). Notably, these serotypes can be further subdivided by the sequence type (ST) by multilocus sequence typing (MLST) (Fig. 1, complete information is shown in Supplementary Table S2). Core genome single nucleotide polymorphism (cgSNP)-based phylogenetic analyses reinforced the result that each serotype includes different lineages. The Japanese O86 isolates could be divided into the following three distinct subgroups: Stx-EAEC O86:H27 ST3570 (JE86-ST02 and 05), STEC O86:H51 ST155 (JE86-ST04), and STEC O86:H32 ST5133 (JE86-ST03). In the first subgroup, two isolates of Stx-EAEC O86:H27 were clustered together with other EAEC O86:H27; however, the other EAEC O86:H27 isolates did not carry *stx2*, except EH3148 (from Belgium in 2018). In the second subgroup (O86:H51 ST155), one Japanese isolate (JE86-ST04) was clustered with three isolates from Japan and the USA. JE86-ST04 possessed the *stx1a* and STEC autoagglutinating adhesin (Saa) gene, which is one of adhesins of locus of enterocyte effacement (LEE)-negative STEC^20^. The other isolates in this cluster did not carry these genes, suggesting that JE86-ST04 could have emerged following their acquisition of the gene in an *stx*-negative O86:H51 strain. The third subgroup was *stx2e*-positive O86:H32 ST5133. The isolates in this subgroup, except FSIS11815123, possessed *stx2e*. Stx2e is a major virulence factor for porcine edema disease^21^ and is rarely associated with human HUS^22^. Although the principal adhesin of edema disease-causing *E. coli,* F18 fimbriae^23^; was not detected, another adhesin for porcine *E. coli*, AIDA (adhesin involved in diffuse adherence)^23^ was detected.

A number of key components involved in the pathogenesis of EAEC have been reported, although not fully understood. The presence and similarity of these virulence factors were compared using draft genomes of EAEC O86:H27 isolates and previously sequenced Stx-EAEC isolates (Supplementary Table S2). The sequences of virulence regulator gene of EAEC (*aggR)*, *aggR*-regulated dispersin gene (*aap*), dispersin transporter operon (*aat*), and type 6 secretion system operon (*aai*), were highly similar (> 95%) among the isolates. In contrast, although all the isolates possessed aggregative adherence fimbriae (AAF), they showed high diversity among the serotypes: O86:H27, AAF/III; O104:H4, AAF/I; O111:H21, AAF/V (Supplementary Table S2).

### Complete genome sequences of Stx-EAEC O86:H27

**Figure 2:**
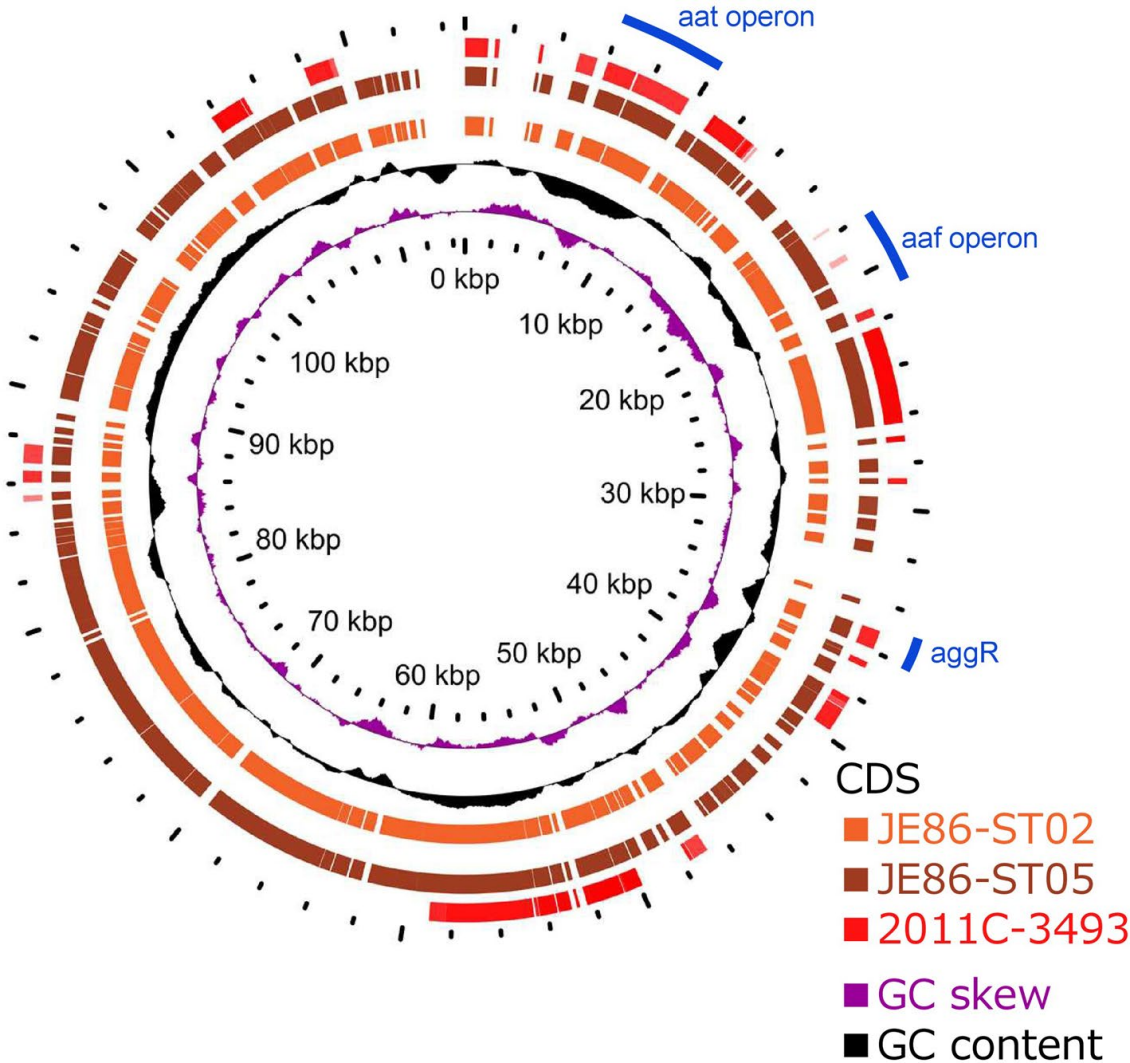
Comparison of the virulence plasmid (pAA) of three isolates of Shiga toxin-producing enteroaggregative *E. coli*. BLAST Atlas analysis was performed by GView Server. A lane for JE86-ST02 represents coding sequences (CDSs) of the pAA. Lanes for JE86-ST05 and 2011C-3493 represent CDSs of pAA that showed more than 80% similarity to the plasmid of JE86-ST02.

By using PacBio and Oxford Nanopore sequencing along with Illumina short read sequencing, complete genome sequences of the Stx-EAEC O86:H27 isolates were determined. Genome statistics are shown in Table 2. Both isolates had a 5.3 Mb chromosome, which is much larger than *E. coli* K12 (ca. 4.7 Mb). PHASTER analysis showed that JE86-ST02 and JE86-ST05 harboured eight and ten putative prophage sequences, respectively. They both had a large plasmid (115 kb) that harboured pAA-borne virulence genes, including *aggR*, an *aaf* gene cluster, *aat*, and an *aap* operon (Fig. 2). Both isolates carried the β-lactamase gene, *bla*_TEM-1B_, in the chromosome, while the epidemic Stx-EAEC O104:H4 strain harbors extended-spectrum β-lactamase genes, including *bla*_CTX-M-15_ and *bla*_TEM-1B_, in an 89 kb- plasmid.

**Table 2:** Statistics of the complete genome sequence.

Statistics	JE86-ST02				JE86-ST05					
	Chromosome	p1	p2	p3	Chromosome	p1	p2	p3	p4	p5
Size (kb)	5,283,470	114,901	83,276	48,397	5,327,513	114,953	85,841	48,666	7,939	5,716
No. CDS	4,996	132	93	71	5,103	134	99	69	9	6
No. rRNA	22	0	0	0	22	0	0	0	0	0
No. tRNA	105	0	0	1	98	0	0	1	0	0
No. prophage-like elements	8	0	0	0	10	0	0	1	0	0
GC%	50.7	48.7	53.1	44.6	50.8	48.7	53.1	44.5	41.6	42.4
Chromosomal sequence type or plasmid Inc type	ST3570	IncFIB, IncFII	IncB/O/K/Z	ND	ST3570	IncFIB, IncFII	IncB/O/K/Z	ND	ND	ND
Antimicrobial resistance gene(s)	*aadA1, ant(3′')-Ia, blaTEM-1B, mdf(A), sul1*	ND	ND	ND	*blaTEM-1B, mdf(A)*	ND	ND	ND	ND	ND
Accession no	AP022811	AP022812	AP022813	AP022814	AP022815	AP022816	AP022817	AP022818	AP022819	AP022820

*ND* not detected.

### Comparative genomics of Stx2a phages and plasmids of Stx-EAEC O86:H27

**Figure 3:**
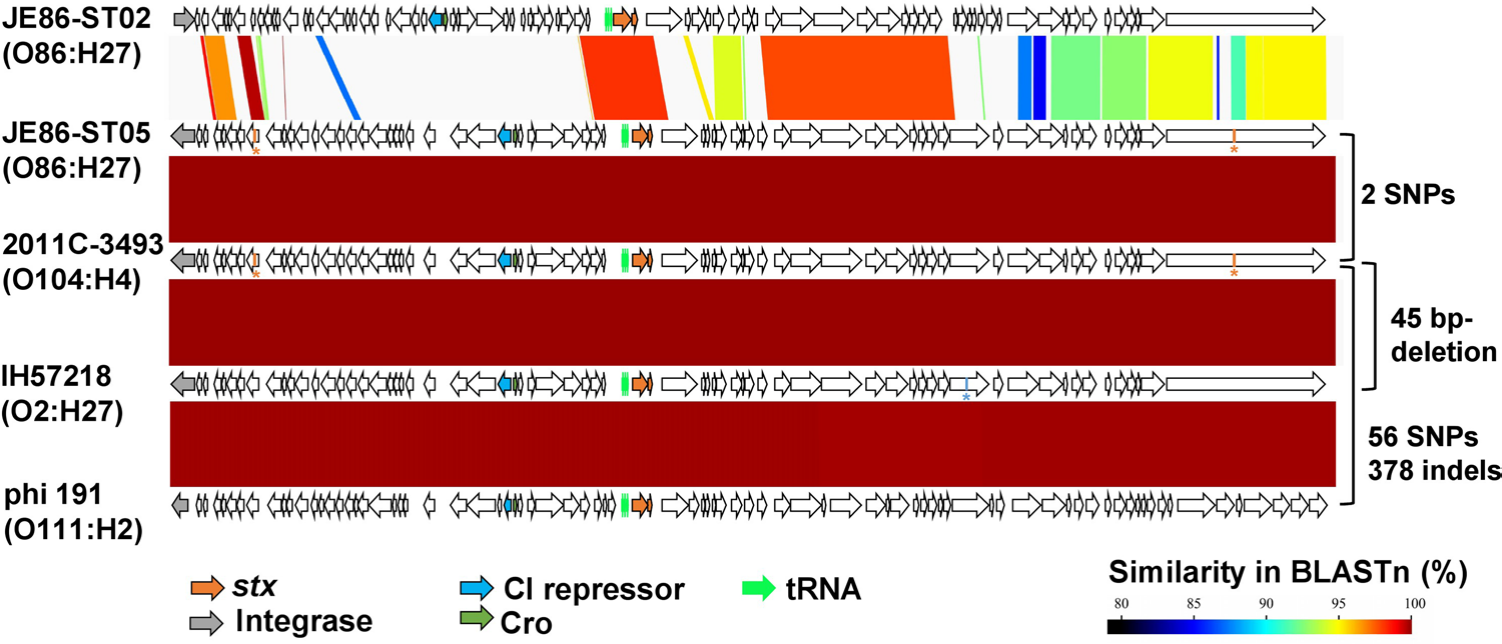
Comparison of the complete sequence of the Shiga toxin (Stx) 2a phage of Stx-enteroaggregative *E. coli* O86:H27, O104:H4, hybrid Shigatoxigenic and enterotoxigenic *E. coli* O2:H27, and O111:H2. Arrows represent the CDSs, and major genes are coloured as shown in the legend. BLASTn and visualization were performed with GenomeMatcher software. The number of single nucleotide polymorphisms (SNPs) and insertions and deletions (indels) to the phage of O104:H4 are shown on the right. Red and blue asterisks show the position of SNPs and deletion, respectively, compared to the phage of O104:H4.

When the complete sequences of the Stx2a phages of JE86-ST02 and JE86-ST05 were compared, only some CDSs in the late region showed similarity (80–99%) (Fig. 3). The integrases of the phages were different and the insertion site of the phages was different (JE86-ST02, *argW*; JE86-ST05, *wrbA*). Interestingly, the results of NCBI-BLAST (https://blast.ncbi.nlm.nih.gov/) showed that the Stx2a phage of JE86-ST05 was highly similar to that of the European epidemic O104:H4 strains (Supplementary Table S4). There were only two loci of SNPs between the phage of JE86-ST05 and that of the European epidemic strain, 2011C-3493 (Fig. 3, Supplementary Table S4). A similar phage from Stx-EAEC O111:H2 (strain ED191, accession no. KF971864) has been reported previously^24^. This strain was the causative agent of the French outbreak in 1992^24,25^. There were 56 SNPs and 378 indels between the O104:H4 2011C-3493 and O111:H2 ED191 phages (Fig. 3). It is of note that the similar phages have been found from other *E. coli* pathotypes. The Stx2a phage from hybrid Shigatoxigenic and enterotoxigenic *E. coli* (STEC/ETEC) isolated from Finland in 2001^26^ had only 45 bp-deletion compared to that of O104:H4 2011C-3493 (Fig. 3). Additionally, the phage of STEC O26 isolates from patients in Europe showed the same phage structure to that of O104:H4 2011C-3493^27^. Meanwhile, similar phages to the Stx2a phage of JE86-ST02 were not found in public databases. Stx2a phages were also found in Stx-EAEC O111:H21 226 from Northern Ireland and Stx-EAEC O86:H27 EH3148 from Belgium. However, their Stx2a phage sequences were quite distinct from both JE86-ST02 and JE86-ST05, according to the draft genome analysis (data not shown).

In contrast, the pAA plasmids of Stx-EAEC O86:H27 are highly similar to each other, but they had low similarity to that of O104:H4 2011C-3493 (Fig. 2). All the coding sequences (CDSs) of JE86-ST02 and JE86-ST05 were shared. The mean ± SD similarity of all CDSs between the two plasmids was 99.9 ± 1.3%.

## Discussion

In this study, we revealed the genomic diversity of domestic and international *E. coli* O86 and found evidence of global distribution of the epidemic-related Stx2a phages.

According to WGS-based phylogeny, the polyphyletic nature of *E. coli* O86 was shown. Serogroup O86 consists of three serotypes (O86:H27, H32, and H51), and each serotype was found in multiple lineages. Even isolates showing different H type clustered together (i.e. in ST101 includes H27 and H51 isolates). This is not surprising because the O- and H-antigen gene clusters can be mobile via recombination^28–30^. Domestic O86 isolates could be divided into three distinct subgroups. Stx-EAEC O86 isolates were clustered with EAEC O86:H27 from Belgium, the UK, and Japan. These isolates shared almost identical virulence components of EAEC, including *aggR*, *aap*, the *aat* operon, T6SS, and SPATEs (Supplementary Table S3), suggesting closely-related EAEC is distributed in Europe and Japan. Although the symptoms caused by the other EAEC O86:H27 isolates were not available, Stx2a might increase the virulence of the bacterium. The other two isolates, JE86-ST03 and 04, were obtained from asymptomatic carriers and thus, their pathogenicity was unclear due to low sample size. JE86-ST03 and JE86-ST04 carried several putative virulence factors of diarrheagenic *E. coli*, including AIDA, *saa*, *cdt*, and *espP*. Further studies are required to elucidate whether these factors may have virulence potential to humans.

Notably, one of the Stx-EAEC O86:H27 isolates harboured an Stx2a phage identical to that of European epidemic Stx-EAEC O104:H4 isolates. A highly similar phage was found in Stx-EAEC O111:H2 19 years before the European epidemic caused by O104:H4^24,25^. The related phages have been found in STEC/ETEC O2:H27 (Finland) and STECs O26 (Europe), providing the evidence of global-level distribution of the Stx2a phage. On the other hand, the two genetically close isolates of Stx-EAEC O86:H27 harboured two distinct Stx2a phages. The two Stx-EAEC isolates, O111:H21 226 from Northern Ireland^6^ and O86:H27 EH3148 from Belgium, also harboured a different Stx2a phage from the other Stx-EAEC. These results provide two implications. First, the epidemic related Stx2a phage is globally distributed among various *E. coli* pathotypes. Second, EAEC has the potential to acquire different Stx2 phages. It is likely that global transfer of the host bacterium facilitates the phage distribution. The principal infection route of EAEC is believed to be human-to-human transmission. Meanwhile, food and animals are indicated to be involved in the transmission of Stx-EAEC O104:H4 and STEC O26^31^, and the related phages were found from cattle^32^. Therefore, various source and transmission routes should be considered to understand the epidemiology of the Stx phage and emergence of highly virulent Stx-EAEC.

Regarding the pathogenicity of EAEC, the pAA and *aggR*-regulated genes have been regarded as key virulence factors in EAEC in vivo and in vitro settings^33,34^, while other report emphasis the importance of certain SPATEs encoded in the chromosome or plasmids^35^.Both Stx-EAEC O86:H27 isolates possessed a set of virulence factors, including, *aggR*, *aggR* regulons, some SPATEs, and other toxins, as other EAEC strains do. Therefore, severe symptoms (e.g. bloody diarrhoea or HUS) by Stx-EAEC O86:H27 may be explained by Stx2a production.

In conclusion, our results indicate the potential of the Stx2a phage of Stx-EAEC O104:H4 to transfer horizontally into phylogenetically distinct strains with few mutations. It is plausible that the Stx2a phage is circulating among various pathotypes of *E. coli*. To understand the epidemiology of the phage in detail, STEC from various locations, sources, pathotypes, and lineages should be considered.

## Methods

### Isolates used in this study

From 1999 to 2017, four STEC O86 isolates were reported through national surveillance (Table 1). Two isolates were from patients, while two isolates were obtained from asymptomatic carriers. These isolates were originally isolated by local health institutes and subsequently sent to our laboratory for further analysis. JE86-ST02 corresponds to 990599 in a previous report by Iyoda et al.^5^.

### WGS phylogeny and in silico typing of draft genomes

Genomic DNA was extracted with the DNeasy Blood & Tissue Kit (QIAGEN, Venlo, Netherlands) and Genomic-tip 100/G (QIAGEN) for short-read and long-read sequencing, respectively. For short-read sequencing, genomic DNA libraries were prepared using a Nextera XT DNA Sample Prep Kit (Illumina, San Diego, CA, USA). The pooled libraries were subjected to multiplexed paired-end sequencing (300-mer × 2) using MiSeq (Illumina). The short reads were assembled using SPAdes v.3.11.1 with the “–careful” option^36^. Contigs of each isolate were comprehensively characterized using an in-house BLAST-based pipeline as described previously^18^. Phylogenetic relationships of STEC O86 isolates and non-O86 *E. coli* belonging to various pathotypes and phylogenetic groups (Supplementary Table S1) were inferred by mapping-based analysis. Core genome SNPs were extracted by using BactSNP v.1.1.0^37^ with the genome of STEC O157 strain Sakai (GenBank accession No.: BA000007) as a reference. Repetitive regions longer than 50 bp were detected by MUMmer v.3.2259 (nucmer, repeat-match, and exact-tandems functions)^38^ and removed for further analyses, as were prophage regions. The recombinogenic regions were detected by gubbins^39^ and removed. Finally, 244,878 SNP sites from 2,074,586 bp of conserved backbone were used for further analyses. Phylogenetic relationships were determined by reconstructing a phylogenetic tree using the maximum likelihood method using IQ-TREE with 1,000 ultrafast bootstrap replicates^40^.

## Determination of the complete genome sequence of Stx-EAEC O86:H27

For the O86:H27 isolates, complete genome sequences were determined by using long read sequencing. For JE86-ST02, PacBio RS II (Pacific Biosciences, Menlo Park, CA) sequencing was performed using the PacBio SMRTbell Template Prep Kit 1.0 and Polymerase Binding Kit P6 after size selection using BluePippin (Sage Science, Beverly, MA) with a cutoff value of 20 kb. The long-read sequences were assembled using the Hierarchical Genome Assembly Process (HGAP) version 3 with SMRT Analysis software^41^ and polished by Pilon^42^ with short-read sequences. For JE86-ST05, a sequencing library was prepared by using a Rapid Barcoding Sequencing Kit (SQK-RBK004, Oxford Nanopore Technologies, Oxford, UK). A MinION R9.4 flow cell (Oxford Nanopore) was used for 48 h sequencing. The long-read sequences and the short-read sequences were subjected to hybrid assembly by using Unicycler v.0.4.4^43^. Annotation for the complete genomes was performed by DFAST^44^ with manual curation. Prophage sequences in the genome were detected by PHASTER^45^. The complete sequences of the pAA plasmids of the O86:H27 isolates were compared to Stx-EAEC O104:H4 2011C-3493 by using GView Server (https://server.gview.ca/). The Stx2a phage sequences of the three isolates used in the plasmid comparison and strain O111:H2 ED191 and O2:H27 IH57218 were compared and visualized by using GenomeMatcher^46^.

### Ethics statement

Patient information of the isolates was completely anonymized.

## Supplementary information

Supplementary file1 (PDF 153 kb)

Supplementary file2 (XLSX 25 kb)

## Data Availability

The complete-genome sequences of JE86-ST02 and JE86-ST05 were submitted to DDBJ/ENA/GenBank under the accession number AP022811-AP022820 as shown in Table 2. Short reads and draft genomes of Japanese *E. coli* O86 isolates were deposited under the accession number DRA007294.
