# Supplementary material for: Global distribution of epidemic-related Shiga toxin 2 encoding phages among enteroaggregative Escherichia coli
Source: Sci Rep. 2020 Jul 16;10:11738. doi: 10.1038/s41598-020-68462-9 (PMC7366661; doi:10.1038/s41598-020-68462-9)
Supplement: Supplementary file 1 — Supplementary file1 (PDF 153 kb) [file 41598_2020_68462_MOESM1_ESM.pdf]

Supplementary information

Global distribution of epidemic-related Shiga toxin 2 encoding phages among enteroaggregative *Escherichia coli*

Keiko Kimata[1], Kenichi Lee[2], Masanori Watahiki[1], Junko Isobe[1], Makoto Ohnishi[2], Sunao Iyoda[2]

[1]Department of Bacteriology, Toyama Institute of Health, Toyama, 9390363, Japan
[2]Deaprtment of Bacteriology I, National Institute of Infectious Diseases, Tokyo, 1628640, Japan

**Table S1**:	Information of publicly available genomic data used in the phylogenetic analysis
**Table S2**:	Comprehensive *in silico* typing of O86 isolates from Japan and EnteroBase (independent Excel file)
**Table S3**:	Relatedness of key virulence determinants of enteroaggregative *Escherichia coli* (EAEC) in Shiga toxin-producing EAEC isolates
**Table S4**:	Results of NCBI-BLAST against the Stx2a phage of JE86-ST05

Table S1. Information of publicly available genomic data used in the phylogenetic analysis

Isolate ID	Serotype/Species	Pathotype	Phylogenetic group	Accession no.	Source	Year	Place
APEC_O1	O1:H7	APEC[a]	B2	CP000468	Poultry	-[b]	USA
12009	O103:H2	STEC	B1	AP010958	Human	2001	Japan
2009EL-2071	O104:H4	Stx-EAEC	B1	CP003301	Human	2009	Republic of Georgia
2009EL-2050	O104:H4	Stx-EAEC	B1	CP003297	Human	2009	Republic of Georgia
2011C-3493	O104:H4	Stx-EAEC	B1	CP003289	Human	2011	USA
11128	O111:H8	STEC	B1	AP010960	Human	2001	Japan
E2348/69	O127:H6	EPEC	B2	FM180568	Human	1969	UK
Sakai	O157:H7	STEC	E	BA000007	Human	1996	Japan
MG1655	O16:H48	Commensal	A	CP025268	-	-	USA
UMN026	O17:H18	ExPEC	D	CU928163	Human	1999	USA
I-151	O174:H46	STEC	E	SAMN02732277	Human	2005	Germany
11368	O26:H11	STEC	B1	AP010953	Human	2001	Japan
042	O44:H18	EAEC	D	FN554766	Human	1983	Peru
S88	O45:H7	ExPEC	B2	CU928161	Human	1999	France
536	O6:H31	UPEC	B2	CP000247	Human	1982	Germany
IAI39	O7:H45	UPEC	F	CU928164	Human	1980s	France
H10407	O78:H11	ETEC	A	FN649414	Human	1973	Bangladesh
LF82	O83:H1	AIEC	B2	CU651637	Human	-	France
290590	O86:H27	EAEC	B1	SRR5470218	Human	2016	UK
300650	O86:H27	EAEC	B1	SRR4788097	Human	2016	UK
ERS1232417	O86:H32	-	E	ERR1789882	-	-	-
AMR520-5149	O86:H32	STEC	A	SRR8079366	Livestock	2014	Canada
AZ-TG60412	O86:H51	-	B1	SRR1186338	Poultry	2013	USA
C00009219	O86:H51	-	A	ERR296688	-	-	-
C309	O86:H32	STEC	A	SRR5927204	Wild Animal	2014	USA
CFS3323	O86:H27	-	B1	ERR3333544	Livestock	2004	Germany
DRS013916	O86:H27	EAEC	B1	DRR015808	-	-	Japan
ECO0117	O86:H51	-	B1	ERR439680	Human	2009	UK
EH3148	O86:H27	Stx-EAEC	B1	Uploaded Reads[c]	-	2018	Belgium
FSIS11815123	O86:H32	-	A	SRR8137335	Livestock	2018	USA
FSIS11919008	O86:H51	-	B1	SRR8754961	Livestock	2019	USA
KS14_4E	O86:H51	-	B1	DRR103021	Livestock	2013	Japan
MOD1-EC6197	O86:H27	-	B1	SRR3465496	Wild Animal	2013	USA
PSU-0601	O86:H51	-	B1	SRR7042103	Wild Animal	1999	USA
PSU-0715	O86:H51	-	B1	SRR7429964	Livestock	2012	USA
TM63	O86:H51	-	E	DRR138475	Human	2018	Thailand
DTU2016_763	O86:H27	-	E	Uploaded Reads	Poultry	2015	Denmark
DTU2017-803	O86:H27	-	E	ERR2091428	Poultry	2016	Denmark
DTU2017-855	O86:H51	-	E	ERR2091353	Poultry	2016	Denmark
ESBL-15/0220	O86:H51	-	B1	ERR2019096	Livestock	2015	Austria
2012EL-1823B	*E. albertii*	-	-	CP030783	Human	2012	USA

[a]APEC, avian pathogenic *E. coli*; STEC, Shiga toxin-producing *E. coli*; Stx-EAEC, Shiga toxin-producing enteroaggregative *E. coli*; EPEC, enteropathogenic *E. coli*; ExPEC, extraintestinal pathogenic *E. coli*; EAEC, enteroaggregative *E. coli*; UPEC, uropathogenic *E. coli*; ETEC, enterotoxigenic *E. coli*; AIEC, adherent invasive *E. coli*.

[b]-, not available.

[c]Uploaded directly to EnteroBase.

Table S3. Relatedness of key virulence determinants of enteroaggregative *Escherichia coli* (EAEC) in Shiga toxin-producing EAEC isolates

Function	Gene	Encoding replicons	O86:H27						O104:H4	O111:H21
			JE86-ST02	JE86-ST05	290590	300650	DRS013916	EH3148	2011C-3493	226
agg regulator	*aggR*	pAA	ref[a]	100	100	100	100	100	96.2	96.2
AAF/III	*aggA*	pAA	ref	100	100	100	100	100	ND[b]	ND[b]
	aggB		ref	100	100	100	100	99.6	ND	ND
	aggC		ref	100	ND	96.3	100	99.8	ND	ND
	aggD		ref	100	100	100	100	100	ND	ND
dispersin and transporter	*aap*	pAA	ref	100	100	100	100	100	96.3	96.3
	aatP		ref	100	100	100	100	99.9	95.0	95.0
	aatA		ref	100	100	100	100	100	95.3	95.5
	aatB		ref	100	100	100	100	100	96.2	96.1
	aatC		ref	100	99.9	100	100	100	96.2	96.2
	aatD		ref	100	100	100	100	100	92.9	92.8
T6SS	*aaiA*	Chromosome	ref	100	100	100	100	100	100	100
	aaiB		ref	100	100	100	100	100	99.9	99.9
	aaiC		ref	100	100	100	100	100	99.8	99.6
	aaiD		ref	100	100	100	100	100	100	100
	aaiE		ref	99.9	99.9	100	100	100	99.9	99.9
	aaiF		ref	100	100	100	100	100	99.9	100
	aaiG		ref	100	100	100	100	100	100	99.9
	aaiH		ref	100	100	100	100	100	100	100
	aaiI		ref	100	100	100	100	100	100	100
	aaiJ		ref	100	100	100	100	100	100	100
	aaiK		ref	100	100	100	100	100	99.8	100
	aaiL		ref	100	100	100	100	100	99.9	99.9
	aaiM		ref	100	100	100	100	100	99.8	99.7
	aaiN		ref	100	100	100	100	100	99.9	99.9
	aaiO		ref	100	100	100	100	99.9	99.9	99.9
SPATEs	*pet*	Plasmid	ND	ND	ND	ND	ND	ND	ND	ND
	pic	Chromosome	ref	100	100	100	100	100	99.9	99.9
	sat	Chromosome	ref	100	100	100	100	100	ND	99.9
	sigA	Chromosome	ND	ref	ND	ND	ND	ND	98.0	ND
	sepA	pAA	ref	100	100	100	100	100	99.6	ND
Biofilm	*fis*	Chromosome	ref	100	100	100	100	100	100	100
	shf	pAA	ref	100	100	100	100	100	98.5	ND
	yafK	Chromosome	ref	100	100	100	100	100	99.5	99.5

[a]Similarity compared to JE86-ST02 or JE86-ST05: ref, reference; number, similarirty %; ND, not detected under the criteria of > 90% length and > 60% identity.

[b]*agg* operon of AAF/III was absent but the *agg* operon of the following subtypes is present: O104:H4, AAF/I; O111:H2, AAF/V.

Table S4. Results of NCBI-BLAST against the Stx2a phage of JE86-ST05

Entry ID[a]	Host serotype	Isolation Year	Isolation country	Epidemiological information	Accession no.	Alignment length (bp)	No. mismatch	No. gaps
FWSEC0009	O104:H4	2011	Germany	Clinical sample	CP031902	60,895	2[bc]	0
LB226692	O104:H4	2011	Germany	HUS patient	CP024992	60,895	2[bc]	0
FDAARGOS_403	O104:H4	Not available	USA	Clinical sample	CP023535	60,895	2[bc]	0
2011C-3493	O104:H4	2011	USA	2011 European outbreak	CP003289	60,895	2[bc]	0
HUSEC2011	O104:H4	2011	Germany	2011 European outbreak	HF572917	60,895	3[bcd]	0
phiON-2011	O104:H4	2011	Canada	2011 European outbreak	KU298437	60,891	2[bc]	0
P13363	O104:H4	2011	Germany	2011 European outbreak	HG803182	60,891	2[bc]	0
FDAARGOS_349	O104:H4	2011	Germany	Human stool	CP027394	60,895	4[bcde]	0
P13374	O104:H4	2011	Germany	2011 European outbreak	HE664024	60,892	4[bcf]	4

[a]Entries that aligned with the full length (> 99%) of the Stx2a phage of JE86-ST05 were shown.

[b]Non-synonymus mutation (V50I) in antirepressor gene.

[c]Non-synonymus mutation (T1674A) in hypothetical gene.

[d]Intergenic region.

[e]Non-synonymus mutation (V136A) in hypothetical gene.

[f]Synonymus mutation in rzpD gene and hypothetical gene.
